# Comprehensive Genomic Investigation of Coevolution of *mcr* genes in *Escherichia coli* Strains via Nanopore Sequencing

**DOI:** 10.1002/gch2.202000014

**Published:** 2021-01-12

**Authors:** Ruichao Li, Pengcheng Du, Pei Zhang, Yan Li, Xiaorong Yang, Zhiqiang Wang, Juan Wang, Li Bai

**Affiliations:** ^1^ Jiangsu Co‐Innovation Center for Prevention and Control of Important Animal Infectious Diseases and Zoonoses College of Veterinary Medicine Yangzhou University Yangzhou 225009 P. R. China; ^2^ Institute of Comparative Medicine Yangzhou University Yangzhou 225009 P. R. China; ^3^ Institute of Infectious Diseases Beijing Ditan Hospital Capital Medical University, and Beijing Key Laboratory of Emerging Infectious Diseases Beijing 100015 P. R. China; ^4^ Key Laboratory of Food Safety Risk Assessment National Health Commission of the People's Republic of China China National Center for Food Safety Risk Assessment Beijing 100022 P. R. China; ^5^ Center for Disease Control and Prevention of Sichuan Province Chengdu 610041 P. R. China; ^6^ College of Veterinary Medicine Northwest A&F University Yangling 712100 P. R. China

**Keywords:** coevolution, colistin resistance, *mcr* genes, plasmids

## Abstract

Horizontal gene transfer facilitates the spread of antibiotic resistance genes, which constitutes a global challenge. However, the evolutionary trajectory of the mobile colistin resistome in bacteria is largely unknown. To investigate the coevolution and fitness cost of the colistin resistance genes in wild strains, different assays to uncover the genomic dynamics of *mcr‐1* and *mcr‐3* in bacterial populations are utilized. *Escherichia coli* strains harboring both *mcr‐1* and *mcr‐3.1/3.5* are isolated and *mcr* genes are associated with diverse mobile elements. Under exposure to colistin, the *mcr‐1*‐bearing resistome is stably inherited during bacterial replication, but *mcr‐3* is prone to be eliminated in populations of certain strains. In the absence of colistin, the persistence rates of the *mcr‐1* and *mcr‐3*‐bearing subclones varies depending on the genomic background. The decay of the *mcr*‐bearing bacterial populations can be mediated by the elimination of *mcr*‐containing segments, large genomic deletions, and plasmid loss. Mobile elements, including plasmids and transposons, are double‐edged swords in the evolution of the resistome. The findings support the idea that antibiotic overuse accounts for global spread of multidrug‐resistant (MDR) bacteria. Therefore, stringent regulation of antibiotic prescription for humans and animals should be performed systematically to alleviate the threat of MDR bacteria.

## Graphical abstract

The evolutionary trajectory of mobile colistin resistance genes and the underlying genomic basis in bacteria are largely unknown after withdrawal of colistin from animal feed additives. This study comprehensively deciphers the genomic landscape after the elimination of *mcr* genes during bacterial growth under different conditions, highlighting the pivotal role of mobile elements in mediating resistance gene transmission.

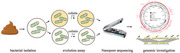

## 1 Introduction

The emergence and persistence of novel antimicrobial resistance genes pose great concern for public health worldwide.^[^ ^1^, ^2^ ^]^ The misuse and abuse of antimicrobials play an important role in the emergence, transmission, and persistence of resistance genes among pathogens, although some resistance genes already existed in the pre‐antibiotic era.^[^ ^3^ ^]^ There are still contrasting opinions concerning the role of clinical antibiotics in the evolution of antibiotic resistance genes, all of which are supported by different facts.^[^ ^4^, ^5^, ^6^ ^]^ The actual correlation between these two factors may depend on the specific resistance mechanisms under investigation, and both ancient origin and post‐antibiotic evolution in different environments may account for the current severe problem of antibiotic resistance in human pathogens. Horizontal gene transfer conferred by plasmids or other mobile elements plays a key role in the transmission of antibiotic resistance among different pathogens.^[^ ^7^ ^]^ Furthermore, the persistence of resistance genes is critical for bacteria to constitute a real ongoing threat. The fitness cost endowed by the acquired resistance genes or plasmids can determine differences in the persistence of resistance genes among bacteria.^[^ ^8^, ^9^ ^]^ However, the role of mobile elements and antibiotics in the persistence and evolution of multiple acquired resistance genes, such as different *mcr* genes, in the same bacterial population has not been extensively investigated.

The emergence of novel plasmid‐associated *mcr* genes (*mcr‐1* to *mcr‐10*) conferring resistance to colistin, a last‐resort antibiotic used to treat severe bacterial infections caused by different pathogens, has evoked great concern for the coming post‐antibiotic era.^[^ ^10^, ^11^, ^12^ ^]^ Co‐occurrence of identical or different *mcr* genes in the same strain was reported.^[^ ^13^, ^14^, ^15^, ^16^ ^]^ The archetypical *mcr‐1*‐bearing composite transposon Tn*6330* was found to be unstable and could lose the *mcr‐1* gene during bacterial replication.^[^ ^17^, ^18^ ^]^ However, the evolutionary trajectory of different *mcr* genes of the same strain during bacterial growth was not investigated systematically. Recently, one report found that plasmids carrying *mcr‐3* were more stable than *mcr‐1*‐bearing plasmids, but the underlying genetic mechanisms were unknown.^[^ ^19^ ^]^ In this study, we comprehensively characterized the genomic basis underlying resistance gene evolution in different *Escherichia coli (E. coli)* strains harboring *mcr‐1* and *mcr‐3* genes. Our findings indicate that the fitness cost of resistance genes and the corresponding plasmids are vital factors affecting the persistence of resistance genes in the bacterial population.

## 2 Results

### 2.1 Characterization of *mcr*‐Bearing *E. coli* Isolates

After bacterial isolation and identification, five *E. coli* strains were found to be positive for both *mcr‐1* and *mcr‐3* and resistant to colistin with minimum inhibitory concentrations ranging from 4 to 8 mg L^−1^. All of them were MDR strains resistant to multiple antimicrobials, including colistin, tetracycline, gentamycin, ampicillin, and chloramphenicol (Table S1, Supporting Information). Conjugation assay was successful for two (CP8‐3 and CP55) out of five strains. For CP8‐3, only the *mcr‐1*‐bearing transconjugant (CP8‐3‐T) was recovered. On the other hand, for CP55 two different transconjugants were obtained (CP55‐T1 harboring *mcr‐1* and CP55‐T2 harboring both *mcr‐1* and *mcr‐3*), indicating that co‐transfer of *mcr‐1* and *mcr‐3* occurred in CP55. The five *mcr*‐bearing strains belonged to different clones with different pulsed‐field gel electrophoresis (PFGE) profiles, implying that these strains descended from various separate ancestors. S1 nuclease digestion PFGE (S1‐PFGE) showed that plasmid profiles differed among strains, ranging from two to four plasmids with different sizes (Figures S1 and S2, Supporting Information). Notably, the plasmid harboring *mcr‐3.19* and *mcr‐1* was previously characterized in an isolate CP53 from the same slaughterhouse.^[^ ^20^ ^]^

### 2.2 Genomic Characterization of *E. coli* Strains Positive for *mcr‐1* and *mcr‐3*

To assess the genomic features of strains harboring both *mcr‐1* and *mcr‐3*, complete genome sequences were successfully obtained using a hybrid de novo assembly strategy. The distribution of resistance genes and basic information on the bacterial genomes are reported in **Table** **1** and Table S2, Supporting Information.

**Table 1:** Genomic features of the five *E. coli* strains positive for the *mcr‐1* and *mcr‐3* genes

Strain	Contigs^a)^	ST	Serotype	Resistance genes	Genetic context of *mcr* genes	Virulence genes	Size (bp)	Plasmid types
CP8‐3	CP8‐3‐chromosome	ST34	–	*mcr‐1.1*, *mdf*(A)	IS*Apl1*‐*mcr‐1*‐*Dpap2*‐IS*1294*‐IS*Apl1;* IS*Kpn19*‐Tn*As2*‐*mcr‐3.5*‐*dgkA*‐IS*15*	*astA*, *gad*	4.7m	chromosome
	pCP8‐3‐IncFII			*aadA1*, *aac(3)‐*VIa, *qnrS1*, *floR*, *tet*(M)			87k	IncFII
	pCP8‐3‐IncFIB			*aadA1*, *aadA2*, *mef*(B), *cmlA1*, *sul3*, *tet*(A), *dfrA12*			75k	IncFIA(HI1), IncFIB(K), IncN
	pCP8‐3‐IncR			*bla*_TEM‐1B_, *mcr‐3.5*, *qnrS1*, *tet*(M)			47k	IncR
	pCP8‐3‐IncX1			–			38k	IncX1
	pCP8‐3‐IncQ			*sul2*			8k	IncQ1
CP55	CP55‐chromosome	ST971	O128:H27	*aac(3)‐IId*, *qnrS1*, *mdf*(A), *floR*, *catA1*	*mcr‐1*‐*pap2*; IS*4321R*‐Tn*As2*‐*mcr‐3.5*‐*dgkA*‐IS*15*	*capU*, *cma*, *gad*, *iha*, *ireA*, *iroN*, *iss*, *lpfA*, *mchF*, *subA*	4.9m	chromosome
	pCP55‐IncFIB			*aph(3″)‐Ib*, *aph(6)‐Id*, *bla*_TEM‐1B_, *sul2*, *tet*(A)			156k	IncFIB, IncFII
	pCP55‐141k			–			141k	IncB/O/K/Z
	pCP55‐IncFII			*mcr‐3.5*, *mph*(A)			70k	IncFII
	pCP55‐IncX4			*mcr‐1.1*			33k	IncX4
CP61	CP61‐chromosome	–	–	*mcr‐1.1*, *mdf*(A)	Tn*6330;* Tn*As2*‐*mcr‐3.1*‐*dgkA*‐IS*Kpn40*	*gad*	4.5m	chromosome
	pCP61‐IncN			*aph(3′)‐Ia*, *qnrS2*, *tet*(A)			80k	ColE10, IncFIA(HI1), IncN,IncR
	pCP61‐IncFIB			*aadA2*, *aadA1*, *aac(3)‐*VIa, *bla*_TEM‐1B_, *mcr‐3.1*, *mef*(B), *cmlA1*, *floR*, *sul3*, *tet*(M), *dfrA12*			92k	IncFIB(K), IncN
CP66‐6	CP66‐6‐chromosome	ST5229	–	*mcr‐1.1*, *mdf*(A)	IS*Apl1*‐*mcr‐1*‐*Dpap2*‐IS*1294*‐IS*Apl1; bla*_TEM‐1B_‐Tn*As2*‐*mcr‐3.5*‐*dgkA*‐IS*26*(IS*15DI*)	*astA*, *gad*, *lpfA*	4.8m	chromosome
	pCP66‐6‐IncFIC			*aadA2*, *aph(3′)‐Ia*, *aadA17*, *aac(3)‐IId*, *aadA1*, *bla*TEM‐215, *lnu*(F), *cmlA1*, *floR*, *sul2*, *sul3*, *tet*(A), *tet*(M), *dfrA12*			99k	IncFIB(AP001918), IncFIC(FII)
	pCP66‐6‐IncFII			*bla*_TEM‐215_, *mcr‐3.5*, *fosA4*, *mph*(A), *dfrA12*			74k	ColE10, IncFII
	pCP66‐6‐IncX3			*bla*_OXA‐181_, *qnrS1*			50k	ColKP3, IncX3
	pCP66‐6‐IncQ			*sul2*			8k	IncQ1
CP131	CP131‐chromosome	ST48	–	*mcr‐1.1*, *mdf*(A)	Tn*6330* (IS*Apl1*‐*mcr‐1*‐*pap2*‐IS*Apl1*); Tn*As2*‐*mcr‐3.1*‐*dgkA*‐IS*Kpn40*	*gad*, *lpfA*	4.5m	chromosome
	pCP131‐IncHI1			*aph(3″)‐Ib*, *aac(3)‐IId*, *bla*_TEM‐1B_, *mcr‐3.1*, *qnrS1*, *mef*(B), *floR*, *sul3*, *tet*(M)			264k	IncFIA(HI1), IncHI1A, IncHI1B(R27)
	pCP131‐IncFIB			*aadA2*, *bla*_TEM‐1B_, *tet*(A), *mph*(A), *floR*, *sul3*, *tet*(M), *dfrA12*			116k	IncFIB(AP001918), IncFIC(FII)

^a)^ All contigs were circular plasmids and chromosomes. Plasmids smaller than 10 kb without resistance genes are not listed here. For the complete sequencing data, refer to the figshare database (https://doi.org/10.6084/m9.figshare.11825871). The dash symbols denote unidentified ST, serotypes, and resistance genes.

**Figure 1:**
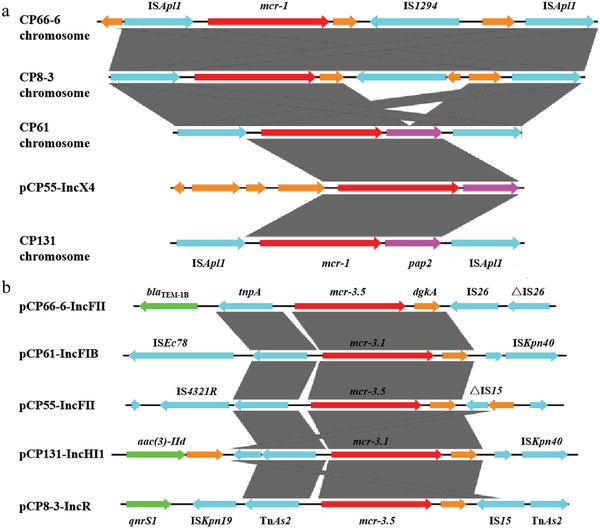
a) Alignment of *mcr‐1*‐bearing DNA segments from the five *E. coli* strains. Red arrows denote the *mcr‐1* gene, while blue arrows represent insertion sequences; yellow and purple arrows are standard for other coding sequences. b) Alignment of *mcr‐3*‐bearing structures of various plasmids. Red arrows denote *mcr‐3* genes, while blue arrows indicate insertion sequences; yellow arrows represent other coding sequences, and green arrows stand for other resistance genes.

The genome of CP8‐3 consisted of one chromosome with the sequence type 34 (ST34) and four plasmids, namely pCP8‐3‐IncFII (87,125 bp), pCP8‐3‐IncFIB (75,733 bp), pCP8‐3‐IncR (47,220 bp), and pCP8‐3‐IncX1 (38,002 bp). The *mcr‐1* gene was located on the chromosome within the genetic structure IS*Apl1*‐*mcr‐1*‐*∆pap2*‐IS*1294*‐IS*Apl1* (**Figure** **1**a). However, the *mcr‐3.5* gene was located on the pCP8‐3‐IncR plasmid within the genetic structure IS*Kpn19*‐Tn*As2*‐*mcr‐3.5*‐*dgkA*‐IS*15*, together with the resistance genes *qnrS1*, *tet*(M), and *bla*_TEM‐1B_. This *mcr‐3.5*‐bearing plasmid was similar to another *mcr‐3*‐bearing plasmid, pHN8 (MG780294), with 99% identity at 83% coverage. The most variable regions between these two plasmids were the MDR regions, showing great diversity mediated by mobile elements (Figure S3, Supporting Information). In addition, pCP8‐3‐IncFII was a typical MDR plasmid including *aadA1*, *aac(3)‐*VIa, *floR*, *tet*(M), and *qnrS1*, and most similar to pCC1410‐1 (KT725788) with 99% identity at 76% coverage. Moreover, pCP8‐3‐IncFIB was a multireplicon MDR plasmid harboring IncFIB and IncN replicons and including *mef(B)*, *sul3*, *cmlA1*, *aadA1*, *dfrA12*, and *tet*(A), and was most similar to pSCE516‐4 (KX023259) with 99% identity at 96% coverage. Finally, pCP8‐3‐IncX1 did not harbor resistance genes, but another plasmid pCP8‐3‐IncQ (8,176 bp) harbored *sul2* and *czcD*, which encoded a heavy metal ion transporter.

The strain CP55 belonged to the ST971 family and carried one chromosome and four plasmids. The gene *mcr‐1* was located on the IncX4‐type plasmid pCP55‐IncX4 (33,309 bp) within the genetic context *mcr‐1*‐*pap2*, without IS*Apl1* flanking *mcr‐1* (Figure 1a). The gene *mcr‐3.5* was located on the IncFII‐type plasmid pCP55‐IncFII (70,770 bp) within the genetic context IS*4321R*‐Tn*As2*‐*mcr‐3.5*‐*dgkA*‐IS*15* (Figure 1b). Another plasmid, pCP55‐IncFIB (156,025 bp), contained multiple resistance genes, including *tet*(A), *sul2*, *strAB*, and *bla*_TEM‐1B_, and was most similar to pH2332‐166 (KJ484626) with 99% identity at 78% coverage.

Furthermore, the strain CP61 harbored one chromosome (untypable ST) and two plasmids, pCP61‐IncFIB (92,073 bp) and pCP61‐IncN (80,920 bp). The gene *mcr‐1* was located on the chromosome in the form of Tn*6330* (IS*Apl1*‐*mcr‐1*‐*pap2*‐IS*Apl1*). However, *mcr‐3.1* was detected in pCP61‐IncFIB in the structure Tn*As2*‐*mcr‐3.1*‐*dgkA*‐IS*Kpn40*, together with *cmlA1*, *aadA1*, *mef(B)*, *sul3*, *bla*_TEM‐1B_, *aac(3)‐*VIa, *floR*, and *tet*(M). The backbone of pCP61‐IncFIB was similar to that of pSCE516‐4 (KX023259) and other reported plasmids (Figure S4, Supporting Information). Finally, the plasmid pCP61‐IncN, carrying multiple replicons, including IncN, IncFIA, IncX1, and IncR, harbored the genes *tet*(A), *aph(3)‐Ia*, and *qnrS2*.

Whole Genome Sequencing (WGS) results showed that in the CP66‐6 strain there was one chromosome (ST5229), harboring IS*Apl1*‐*mcr‐1*‐*∆pap2*‐IS*1294*‐IS*Apl1*, and four plasmids, namely pCP66‐6‐IncFIC (99,734 bp), pCP66‐6‐IncFII (74,817 bp), pCP66‐6‐IncX3 (50,481 bp), and pCP66‐6‐IncQ (8,197bp), invisible by S1‐PFGE. pCP66‐6‐IncFIC harbored IncFIC and IncFIB replicons and included the genes *tet*(M), *aadA1*, *cmlA1*, *aadA1*, *dfrA12*, *floR*, *sul2*, *tet*(A), *aph(3*′*)‐Ia*, *sul3*, *bla*_TEM‐1B_, *lnu(F)*, *aadA1*, and *aac(3)‐IId*. In addition, pCP66‐6‐IncFII harbored *mcr‐3.5* in the core structure *bla*_TEM‐1B_‐Tn*As2*‐*mcr‐3.5*‐*dgkA*‐IS*26*(IS*15DI*), together with *mph(A)*, *dfrA12*, and *fosA*. This *mcr‐3*‐bearing plasmid was most similar to pCHL5009T‐102k‐mcr3 (CP032937), with 99% identity at 74% coverage (Figure S5, Supporting Information). The third plasmid, pCP66‐6‐IncX3, harbored *bla*_OXA‐181_, *qnrS1*, and was nearly identical to pM206‐OXA181(AP018831) at 100% coverage. Finally, pCP66‐6‐IncQ was nearly identical to pCP8‐3‐IncQ, with only a few SNPs.

The CP131 strain consisted of one chromosome (ST48) and two MDR plasmids, namely pCP131‐IncHI1 (264,177 bp) and pCP131‐IncFIB (121,655 bp). The *mcr‐1* gene was located on the chromosome in the form of Tn*6330*. The *mcr‐3.1* gene was found in pCP131‐IncHI1 in the structure Tn*As2*‐*mcr‐3.1*‐*dgkA*‐IS*Kpn40* (Figure 1), together with other resistance genes, including *floR*, *aac(3)‐IId*, *strAB*, *mef(B)*, *sul3*, *qnrS1*, and *bla*_TEM‐1B_. Finally, pCP131‐IncFIB harbored *tet*(A), *mph(A)*, *floR*, *bla*_TEM‐1B_, and *tet*(M).

### 2.3 Comparison of *mcr*‐Bearing Mobile Elements and Detection of Circular Intermediates

Across the five strains, *mcr‐1* was found on the chromosomes of CP8‐3, CP61, CP66‐6, and CP131 and on a plasmid of CP55 (Table S3, Supporting Information). An intact Tn*6330* structure was found in the chromosomes of CP61 and CP131, while a Tn*6330* truncated by the insertion of IS*1294* (IS*Apl1*‐*mcr‐1*‐*∆pap2*‐IS*1294*‐IS*Apl1*) was observed in both CP8‐3 and CP66‐6. However, the insertion sites of *mcr‐1* on the chromosomes varied, consistent with previous reports.^[^ ^21^ ^]^ In contrast, in CP55 the *mcr‐1* gene was located on the IncX4‐type plasmid pCP55‐33k, which has a structure similar to that of other reported *mcr‐1*‐bearing IncX4 plasmids.

**Figure 2:**
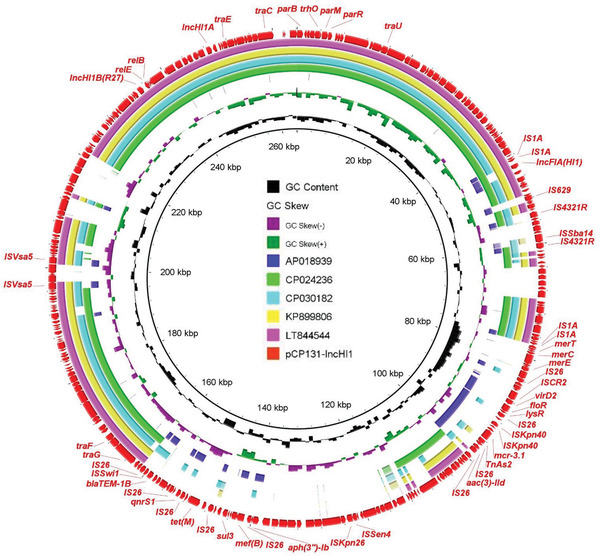
Alignment of the novel *mcr‐3.1*‐bearing plasmid pCP131‐IncHI1 with other similar plasmids. The outermost circle with arrows denotes the reference plasmid pCP131‐IncHI1.

On the other hand, in the five strains, *mcr‐3.1* or *mcr‐3.5* was located on plasmids harboring IncFII, IncR, IncHI1, or IncFIB replicons, with sizes ranging from 47 to 264 kb. Although the plasmids and surrounding sequences were diverse, the core *mcr‐3*‐containing structure Tn*As2*‐*mcr‐3*‐*dgkA*, flanked by mobile elements or resistance genes, was conserved (Figure 1b). The *mcr‐3.1*‐bearing plasmid pCP131‐IncHI1 was an MDR IncHI1/IncFIA‐type plasmid containing multiple resistance genes (**Figure** **2**). To the best of our knowledge, this is the largest *mcr‐3.1*‐bearing plasmid ever reported.

To investigate the transmission of *mcr* genes, reverse PCR was used to detect potential circular intermediates according to published methods.^[^ ^22^ ^]^ Samples positive for Tn*6330* or its variant (IS*Apl1*‐*mcr‐1*‐*∆pap2*‐IS*1294*‐IS*Apl1*) were identified with the detection of two types of PCR products (2.5 and 4 kb, respectively). Sanger sequencing confirmed the existence of two types of circular forms, IS*Apl1*‐*mcr‐1*‐*pap2* and IS*Apl1*‐*mcr‐1*‐*∆pap2*‐IS*1294*. In addition, two types of circular intermediates of *mcr‐3.1*, producing PCR products of 2 kb (CP66‐6) and 4.5 kb (CP131), were identified and sequencing analysis demonstrated that the two complete circular structures were ∆IS*26*‐Tn*As2*‐*mcr‐3.5*‐*dgkA* and ∆IS*26*‐∆Tn*As2*‐*mcr‐3.1*‐*dgkA*‐IS*Kpn40*‐*ble*. These results imply that IS*Apl1* and IS*26* play pivotal roles in the formation of circular intermediates of *mcr‐1* and *mcr‐3*, respectively. It has been reported that *mcr‐1*‐bearing circular intermediates may be derived from an excision mechanism.^[^ ^23^, ^24^ ^]^ In contrast, *mcr‐3* circular intermediates may be formed and translocated to other loci by a copy‐and‐paste mechanism, since with PCR primers targeting flanking sequences of the core *mcr‐3*‐bearing region we did not detect an excised region (data not shown).

### 2.4 Coevolution of *mcr* Genes and Underlying Genomic Dynamics

To investigate the coevolution of *mcr‐1* and *mcr‐3* genes in the same *E. coli* strain, we performed a stability assay of *mcr* genes in the five strains, as well as in the previously reported CP53 strain.^[^ ^13^, ^18^ ^]^ Under colistin pressure, the *mcr‐1* gene was stable in all five strains and no *mcr‐1*‐negative subclones were detected during serial culture steps except for strain CP131, in which twelve subclones at the culture day 28 were found to be negative for *mcr‐1* but still positive for *mcr‐3* (Table S4, Supporting Information). After serial passages in medium with colistin, loss of *mcr‐3* genes was detected in CP61 and CP66‐6, among which CP66‐6 lost *mcr‐3* most easily, with 100% *mcr‐3*‐negative bacterial clones at passage 28 (Table S4, Supporting Information). No clone was observed to have lost both *mcr‐1* and *mcr‐3* under colistin pressure. However, loss of *mcr‐1* and/or *mcr‐3* in absence of colistin was common. Indeed, *mcr‐1* was consistently stable only in CP55 along serial passages over 14 days. On the other hand, CP66‐6 and CP61 easily lost *mcr‐1* and/or *mcr‐3* after a number of passages. Intriguingly, all the *mcr‐1*‐negative clones of CP66‐6 were also negative for *mcr‐3*. To further investigate the genetic background and molecular mechanisms leading to loss of *mcr‐1* and/or *mcr‐3*, we performed S1‐PFGE of subclones and obtained complete sequences of *mcr*‐bearing plasmids or chromosomes.

**Figure 3:**
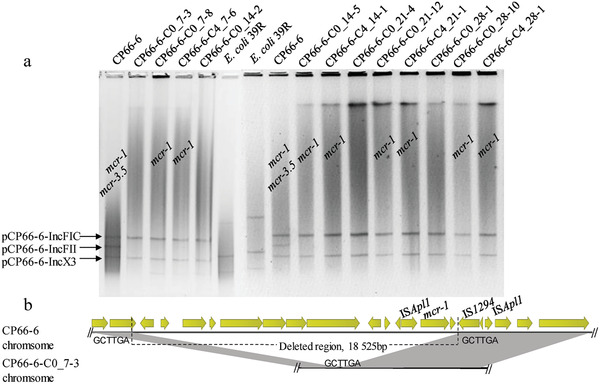
a) Results of S1‐PFGE for CP66‐6 and its subclones after serial passages in colistin and colistin‐free medium. The lanes of *mcr*‐positive clones are labeled. C0 denotes clones grown in medium without colistin, while C4 is used for clones grown with 2 mg L^−1^ colistin. The numbers 7, 14, 21, and 28 indicate the passage number (one passage per day) at which the clone is isolated. The last number indicates the clone number in the plates screened for *mcr* genes. b) Sequence alignment between the *mcr‐1*‐bearing chromosomal region of CP66‐6 and the corresponding chromosomic region of the subclone CP66‐6‐C0_7‐3.

In CP66‐6, the extinction of *mcr‐3* was caused by the loss of the *mcr‐3*‐bearing plasmid pCP66‐6‐IncFII, according to the S1‐PFGE profile (**Figure** **3**a). However, it was impossible to assess the cause of the loss of chromosomal *mcr‐1* by S1‐PFGE. To address the knowledge gap, the subclone CP66‐6‐C0_7‐3 was selected for WGS by MinION long‐read sequencing. Three circular contigs, including one chromosome and two plasmids (pCP66‐6‐IncFIC and pCP66‐6‐IncX3), were assembled. No *mcr‐3*‐bearing plasmid was identified, consistent with the S1‐PFGE results (Figure 3a). Detailed analysis of the *mcr‐1*‐bearing chromosomal segment indicated that an 18‐kb region including IS*Apl1*‐*mcr‐1*‐*pap2* was deleted in the chromosome of CP66‐6‐C0_7‐3 (Figure 3b). IS*1294* may be involved in the deletion of this region. However, further research is required to reveal the underlying molecular mechanism. In conclusion, we propose that *mcr‐1*‐negative CP66‐6 subclones are derived from the deletion of chromosomal *mcr‐1* during bacterial replication.

Regarding the subclones of CP8‐3, the extinction of *mcr‐3.5* in CP8‐3‐C0_1‐7 resulted from the loss of the *mcr‐3.5*‐bearing plasmid pCP8‐3‐IncR, as shown by S1‐PFGE and confirmed by MinION sequencing (Figure S4a, Supporting Information). In both CP8‐3‐C0_21‐5 and CP8‐3‐C0_28‐1, whose parent strain CP8‐3 displayed the same core *mcr‐1*‐bearing structure IS*Apl1*‐*mcr‐1*‐*∆pap2*‐IS*1294*‐IS*Apl1* as CP66‐6, the loss of chromosomal *mcr‐1* was due to the deletion of a long chromosomal region extending beyond the IS*Apl1* boundaries, similar to that observed in CP66‐6‐C0_7‐3. This was partially confirmed by the absence of PCR products when targeting the proximal genes of the core *mcr‐1*‐bearing structure.

**Figure 4:**
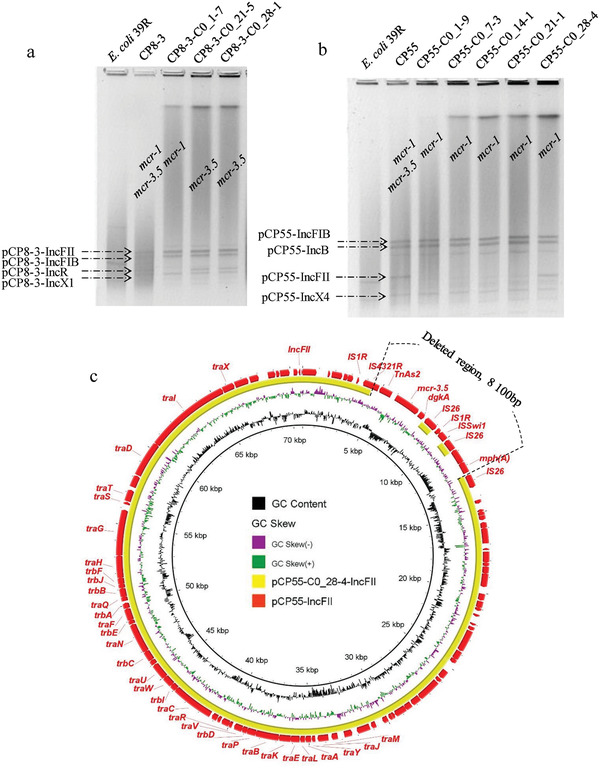
a,b) Results of S1‐PFGE of CP8‐3, CP55, and their respective subclones after serial passages in colistin and colistin‐free medium. c) Circular sequence alignment between the original *mcr‐3.5*‐bearing pCP55‐IncFII plasmid and its derivatives displaying loss of *mcr‐3.5*. The outermost circle represents the reference plasmid pCP55‐IncFII.

No subclones losing *mcr‐1* were found for the CP55 strain. However, *mcr‐3.5* extinction in CP55 subclones may have resulted from the loss of the *mcr‐3.5*‐bearing segment of the pCP55‐IncFII plasmid, since the *mcr‐3.5*‐negative subclone CP55‐C0_28‐4 pCP55‐IncFII became smaller (**Figure** **4**b). To confirm this hypothesis, the complete plasmids of CP55‐C0_28‐4 were sequenced by MinION sequencing. The comparison between the plasmids pCP55‐IncFII and pCP55‐C0_28‐4‐IncFII showed that an 8‐kb region including IS*4321R*‐Tn*As2*‐*mcr‐3.5*‐*dgkA*‐IS*15‐mph(A)* was deleted in pCP55‐C0_28‐4‐IncFII (Figure 4c). We speculate that the loss of the *mcr‐3.5*‐bearing region of pCP55‐IncFII may have also generated the other four *mcr‐3.5*‐negative subclones of CP55.

**Figure 5:**
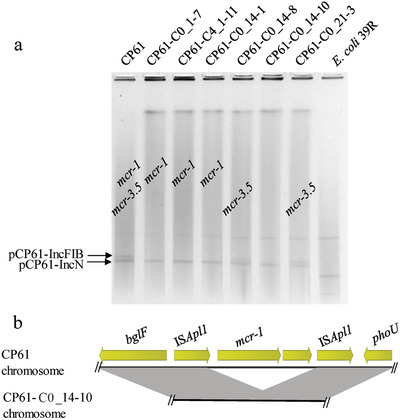
a) Results of S1‐PFGE of CP61 and its subclones after serial passages in colistin and colistin‐free medium. b) Linear alignment between the *mcr‐1*‐bearing chromosomic region of CP61 and its truncated version from the subclone CP61‐C0_14‐10.

**Figure 6:**
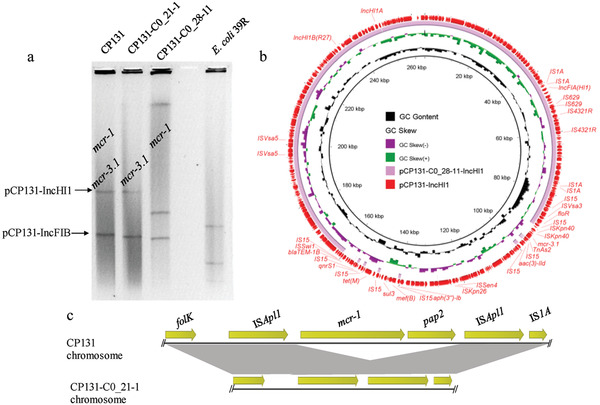
a) Results of S1‐PFGE of CP131 and its subclones after serial passages in colistin and colistin‐free medium. b) Comparison of pCP131‐IncHI1 and the respective plasmid from the subclone CP131‐C0_28‐11, without *mcr‐3.1*. c) Comparison of the *mcr‐1*‐bearing chromosome of CP131 and its evolved version from the subclone CP131‐C0_21‐1.

Furthermore, the loss of the *mcr‐3.5*‐bearing plasmid pCP61‐IncFIB accounted for the loss of *mcr‐3.5* in CP61 subclones (**Figure** **5**a). In addition, according to Nanopore long‐read data, chromosomal *mcr‐1* loss in CP61 subclones was due to the loss of IS*Apl1*‐*mcr‐1*‐*pap2*, resulting in one residual IS*Apl1* sequence (Figure 5b). This represents the typical decay process of Tn*6330*.^[^ ^17^ ^]^ As for CP131, two subclones negative for *mcr‐1* or *mcr‐3.5* were analyzed by S1‐PFGE (**Figure** **6**a). Notably, the extinction of *mcr‐3.5* in CP131‐C0_28‐11 was due to the deletion of a large region in the pCP131‐IncHI1 plasmid mediated by IS*15* (IS*26*‐like), as confirmed by the comparison between whole plasmid sequences (Figure 6b). In the CP131‐C0_21‐1 subclone, deletion of chromosomal *mcr‐1*‐*pap2*, leaving two residual IS*Apl1* sequences in the chromosome, accounted for the loss of *mcr‐1* (Figure 6c). This was inconsistent with our results related to CP61 subclones and our previous report,^[^ ^17^ ^]^ which found that Tn*6330* decay would result in one residual IS*Apl1* sequence. The reason underlying this difference requires further research.

## 3 Discussion

The emergence of the mobile colistin resistance gene *mcr* was traced back to the 1980s,^[^ ^25^ ^]^ indicating that the spread of *mcr‐1* and its alleles among different pathogens is a long‐lasting evolutionary event. Mobile elements, antibiotic pressure, fitness cost, and compensatory mutations are pivotal factors for *mcr* gene transfer and persistence in the microbiome from different habitats, including animals, humans, and the environment. The expression of *mcr‐1* could confer a biological fitness cost in different bacterial species, including *E. coli* and *Klebsiella pneumoniae*.^[^ ^8^, ^26^, ^27^ ^]^ However, the fitness cost of other *mcr* genes has not been investigated.^[^ ^19^ ^]^ Recently, co‐occurrence of different *mcr* genes was reported in single strains of *E. coli*.^[^ ^13^, ^14^, ^19^, ^28^ ^]^ Nevertheless, the possible coevolution of *mcr‐1* and *mcr‐3.1*/*3.5* has not been previously investigated.

In this study, we found five *E. coli* strains of different STs to be positive for *mcr‐1* and *mcr‐3.1*/*3.5*. Three strains harbored *mcr‐1* and *mcr‐3.5*, while two harbored *mcr‐1* and *mcr‐3.1*. The co‐occurrence of *mcr‐1* and *mcr‐3.5* was consistent with previous reports.^[^ ^19^, ^29^ ^]^ The *mcr‐1* gene could be found in both plasmids and chromosomes.^[^ ^21^, ^23^, ^30^ ^]^ However, various *mcr‐3* genes were only found in different Inc‐type plasmids in bacteria, except in *Aeromonas* species, which are potential reservoirs of *mcr‐3* genes.^[^ ^31^, ^32^, ^33^ ^]^ This phenomenon possibly explains the lower occurrence of *mcr‐3* than *mcr‐1* in field strains isolated from different sources.^[^ ^29^ ^]^ Indeed, unlike the typical *mcr‐1*‐bearing Tn*6330*,^[^ ^21^ ^]^ the core genetic context of *mcr‐3* may also limit its transfer ability to the chromosomes of Enterobacteriaceae. The expression of *mcr‐3* was reported to be less costly than that of *mcr‐1* in the laboratory *E. coli* strain TOP10, with *mcr‐3*‐bearing plasmids outnumbering *mcr‐1*‐bearing plasmids in bacterial populations of this strain.^[^ ^19^ ^]^ Although some strains in this study followed this paradigm, exceptional strains such as CP66‐6 showed the opposite trend, implying that the fitness cost of different *mcr* genes could depend on the bacterial genomic background rather than on the genes themselves. Furthermore, a previous study also demonstrated that the ratio of *mcr‐3*/*mcr‐1* plasmids consistently increased over time in all three tested wild strains both in the presence and absence of colistin.^[^ ^19^ ^]^ However, the data in this study showed that *mcr‐3* genes were more prone to be eliminated than *mcr‐1* during serial passages of certain bacterial strains (e.g., CP66‐6), with or without colistin. This indicated that, apart from the properties of *mcr* genes themselves, the genetic background, including diverse plasmids and chromosomes, may be important for determining their evolutionary trajectory. Therefore, the fitness cost of different *mcr* genes could depend on the genomic landscape, and direct comparison of fitness cost of resistance genes between different bacterial species, or even between various clones of the same species, should be conducted with caution. Indeed, plasmid‐host adaptation could influence the destiny of MDR plasmids in pathogens.^[^ ^9^, ^34^ ^]^ Moreover, in addition to single bacteria, biofilm formation is also a factor influencing the coevolution of resistance genes, plasmids, and their hosts.^[^ ^35^ ^]^

Certain widespread *mcr‐1*‐bearing plasmids (IncX4 and IncI2) did not confer fitness cost during bacterial growth under no colistin pressure.^[^ ^36^ ^]^ However, although *mcr‐1*‐bearing plasmids could stably persist in bacteria, the *mcr‐1*‐bearing composite transposon Tn*6330* was dynamic and loss of *mcr‐1* mediated by IS*Apl1* could occur.^[^ ^17^, ^18^ ^]^ In our study, detailed genetic analysis of the passaged subclones showed that *mcr‐1* was more stable than *mcr‐3* in the strains harboring these two genes under antibiotic exposure, and loss of *mcr‐1* was mediated by Tn*6330* decay or deletion of large genomic regions. In contrast, loss of *mcr‐3*‐bearing plasmids and partial deletion of plasmid regions mediated by insertion sequences were two major reasons for *mcr‐3.1*/*3.5* deletion. This indicates that *mcr‐3*‐bearing plasmids may confer greater fitness cost than *mcr‐1*‐bearing plasmids and chromosomes, which is consistent with the fact that *mcr‐3*‐bearing plasmids are less frequent than *mcr‐1*‐bearing plasmids among the *mcr*‐bearing strains.^[^ ^37^ ^]^

## 4 Conclusions

In conclusion, this study demonstrated that the presence of colistin provided the vital driving force for the retention of *mcr* genes in bacteria and that the co‐occurrence of multiple *mcr* genes was not an optimal evolutionary event under no exposure to colistin. Deletion of resistance regions and plasmid loss were two genetic events that the bacteria utilized to eliminate *mcr‐3* genes during growth. On the other hand, DNA fragment deletion mediated by mobile elements was the major cause of *mcr‐1* elimination. These results support the practices of banning the usage of colistin in veterinary feed additives and optimizing colistin deployment in clinical settings worldwide. This study expands the understanding of the coevolution of novel mobile resistance genes in bacterial populations. The long‐term coevolution of multiple *mcr* genes in bacterial communities containing different bacterial species or clones requires further detailed investigations.

## 5 Experimental Section

##### Isolation of Bacterial Strains

A pig slaughterhouse in Sichuan, China was selected to isolate colistin‐resistant *E. coli* strains in July 2016. Briefly, pig feces were collected in sterile sampling bags, stored at low temperature (4 °C), and transported to the laboratory for bacterial isolation. The feces (0.5 g) were mixed with 1 mL sterile saline solution (0.9% NaCl) and 50 µL of this fecal solution was spread on MacConkey agar supplemented with 4 µg mL^−1^ colistin. Putative *E. coli* strains appearing as red colonies were selected for bacterial identification (six colonies per sample). The VITEK2 COMPACT instrument and 16S rRNA gene sequencing were used to identify bacterial species. Pure bacterial cultures were stored in 15% glycerol at −80 °C.

##### Identification of mcr‐1‐ and mcr‐3‐Bearing Strains, Conjugation Assay, PFGE, and S1‐PFGE

Bacterial strains identified as *E. coli* were screened via PCR with primers targeting *mcr‐1* and *mcr‐3* (MCR‐1F, ATCAGCCAAACCTATCCTATCG; MCR‐1R, ATAGATGTTGCTGTGCGTCTGC; MCR‐3F, TATGGGTTACTATTGCTGG; MCR‐3R, CGATGAGCATCAGGGTAG). All strains harboring *mcr‐1* and *mcr‐3* were typed by the PFGE method for bacterial genomes after *Xba*I digestion, with the genome of the *Salmonella* Braenderup strain H9812 as standard marker. A conjugation assay through the filter mating method was conducted to test the transfer ability of colistin resistance genes with *E. coli* J53 (Azi^r^) as the recipient strain. To visualize the plasmid profiles of the original isolates and their transconjugants, PFGE of bacterial genomes after S1 nuclease digestion (S1‐PFGE) was performed. Antimicrobial susceptibility testing with sixteen drugs was performed using the microbroth dilution method (Table S1, Supporting Information) and results were interpreted according to the CLSI Standard.^[^ ^38^ ^]^ *E. coli* ATCC 25 922 was used as quality control.

##### WGS and Bioinformatic Analysis

Genome sequencing utilizing short‐read Illumina and long‐read MinION platforms were performed to obtain the complete genomic sequences of *E. coli* strains harboring *mcr‐1* and *mcr‐3* according to a published method.^[^ ^39^ ^]^ Briefly, paired‐end short reads (2 × 150 bp) were obtained by Illumina Hiseq 2500 sequencing, long reads were generated with the Rapid Barcoding Kit (SQK‐RBK004) and flowcell R9.4 in a MinION sequencer, and hybrid de novo assembly was performed with Unicycler.^[^ ^40^, ^41^ ^]^ Total genomes or plasmids of passaged bacterial populations were sequenced with a long‐read MinION platform and assembled using the Flye or Canu tools.^[^ ^42^, ^43^ ^]^ Circular complete chromosome and plasmid sequences were annotated by Rapid Annotation using Subsystem Technology and edited manually.^[^ ^44^ ^]^ Different databases, including ISFinder, PlasmidFinder, and ResFinder, were utilized to investigate the detailed structures of genomes.^[^ ^45^, ^46^, ^47^ ^]^ The BRIG and Easyfig tools were used to perform genetic context comparisons.^[^ ^48^, ^49^ ^]^

##### Coevolution Assay of mcr‐1 and mcr‐3 in E. coli Populations

To evaluate the stability and evolutionary dynamics of the colistin resistance genes *mcr‐1* and *mcr‐3*, serial passages of the *E. coli* strains CP8‐3, CP55, CP61, CP66‐6, and CP131, harboring both *mcr‐1* and *mcr‐3* genes, were performed as follows: The five strains were grown on TSA agar plates supplemented with colistin (2 µg mL^−1^) and the same single colony from each strain was inoculated into 10 mL LB broth with colistin (2 µg mL^−1^) or without antibiotics and placed in an incubator at 37 °C with shaking at 100 rpm. Then, 20 µL bacterial cultures were transferred (passage 1) onto 10 mL fresh broth (1:500 dilution) with the same conditions and incubated overnight at 37 °C with shaking at 100 rpm. After incubation, these cultures were diluted (passage 2) and 100 µL of the dilutions were spread on TSA agar; after incubation, twelve colonies from a quarter region of a plate were selected to perform genomic DNA isolation and PCR‐based detection of *mcr‐1* and *mcr‐3* to investigate the frequency of *mcr* gene loss in the bacterial populations. The cultures were then passaged for seven days based on the method described above. After passage 7, the cultures were again used to investigate the frequency of *mcr* gene loss. The same procedure was then performed after passage (day) 14, 21, and 28. In addition, S1‐PFGE was utilized to investigate differences in plasmid profiles among subclones of the same strain during different stages of serial culture.

##### Detection of Circular Intermediates

To investigate the potential role of circular intermediates in mediating the transfer of *mcr‐1* and *mcr‐3* genes, reverse primers (MCR1‐RC‐F, ACGCACAGCAATGCCTATGA; MCR1‐R, CTTGGTCGGTCTGTAGGG; MCR3‐cF, CCGTGTTCCTATGCAGGTGT; MCR3‐cR, GAGAACTCCACGCCAGTTCA) were designed and long‐range PCR was performed to test the potential presence of circular DNA forms.^[^ ^22^ ^]^ PCR products were then sequenced using the Sanger method.

## Conflict of Interest

The authors declare no conflict of interest.

## Availability of Data and Materials

The genome sequences generated in this study were deposited in NCBI with the BioProject number PRJNA633463. The data were also deposited in the figshare database (https://doi.org/10.6084/m9.figshare.11825871) for reference.

## Supporting information

Supporting Information
