# Supplementary material for: Comprehensive Genomic Investigation of Coevolution of mcr genes in Escherichia coli Strains via Nanopore Sequencing
Source: Glob Chall. 2021 Jan 12;5(3):2000014. doi: 10.1002/gch2.202000014 (PMC7933819; doi:10.1002/gch2.202000014)
Supplement: Supplementary file 1 — Supporting Information [file GCH2-5-2000014-s001.pdf]

© 2021 Wiley-VCH GmbH

Global Challenges Open Access

Supporting Information

for *Global Challenges,* DOI: 10.1002/gch2.202000014

Comprehensive Genomic Investigation of Coevolution of *mcr* genes in *Escherichia coli* Strains via Nanopore Sequencing

*Ruichao Li, Pengcheng Du, Pei Zhang, Yan Li, Xiaorong Yang, Zhiqiang Wang, Juan Wang, and Li Bai**

Supplementary Data

Comprehensive genomic investigation of coevolution of *mcr* genes in *E. coli* strains via nanopore sequencing

Ruichao Li[1,2]†, Pengcheng Du[3]†, Pei Zhang[4], Yan Li[1], Xiaorong Yang[5], Zhiqiang Wang[1], Juan Wang[6], Li Bai[4]*

[1] Jiangsu Co-Innovation Center for Prevention and Control of Important Animal Infectious Diseases and Zoonoses, College of Veterinary Medicine, Yangzhou University, Yangzhou, Jiangsu, P. R. China;

[2] Institute of Comparative Medicine, Yangzhou University, Yangzhou, Jiangsu, P. R. China;

[3] Institute of Infectious Diseases, Beijing Ditan Hospital, Capital Medical University, and Beijing Key Laboratory of Emerging Infectious Diseases, Beijing, P. R. China;

[4] Key Laboratory of Food Safety Risk Assessment, National Health Commission of the People's Republic of China, China National Center for Food Safety Risk Assessment, Beijing, P. R. China;

[5] Center for Disease Control and Prevention of Sichuan Province, Chengdu, Sichuan, P. R. China;

[6] College of Veterinary Medicine, Northwest A&F University, Yangling, Shaanxi, P. R. China.

Running title: Characterization of bacterial populations harboring *mcr-1* and *mcr-3* genes

†Contributed equally to the work

***Corresponding author**: Li Bai, Tel: 8610-52165543; Email: baili@cfsa.net.cn

China National Center for Food Safety Risk Assessment, Beijing, China

Key words: colistin resistance, plasmids, *mcr* genes, coevolution

Table S1. Antimicrobial susceptibility testing of different *E. coli* strains and their transconjugants against different drugs (MICs, μg/mL).

Strains[a]	Antimicrobials[b]														
	CTX	CAZ	IMP	TET	CFZ	CFX	GEN	AMP	AMS	CHL	CIP	SXT	NAL	AZI	CT
CP8-3	≤0.25	≤1	≤0.25	>32	2	8	>32	>64	8	>64	1	>8/152	16	16	8
CP8-3-T	≤0.25	≤0.5	≤0.25	32	4	8	32	>64	8/4	>64	0.5	≤0.25/4.75	16	≤2	4
CP55	≤0.25	≤0.5	≤0.25	>32	4	8	16	>64	8/4	>64	0.25	≤0.25/4.75	8	≤2	8
CP55-T1	≤0.25	≤0.5	≤0.25	4	4	16	≤1	8	8/4	8	≤0.03	≤0.25/4.75	≤4	≤2	4
CP55-T2	≤0.25	≤0.5	≤0.25	4	2	4	≤1	4	4/2	8	≤0.03	≤0.25/4.75	≤4	4	4
CP61	≤0.25	≤1	≤0.25	>32	4	8	>32	>64	8	>64	0.5	>8/152	8	8	4
CP66-6	0.5	≤1	1	>32	16	32	>32	>64	>64	>64	32	>8/152	>64	>64	8
CP131	≤0.25	≤1	≤0.25	>32	8	16	>32	>64	16	>64	2	>8/152	>64	16	4

[a] The strains with the letter T were the corresponding transconjugants with *E. coli* J53 as the recipient strain.

[b] Drugs abbreviations: CTX, cefotaxime; CAZ, ceftazidime; IMP, imipenem; TET, tetracycline; CFZ, cefazolin; CFX, cefoxitin; GEN, gentamycin; AMP, ampicillin; AMS, ampicillin/sulbactam; CHL, chloramphenicol; CIP, ciprofloxacin; SXT, trimethoprim/sulfamethoxazole; NAL, nalidixic acid; AZI, azithromycin; CT, colistin.

Table S2. Distribution of resistance genes, insertion sequences and replicon genes among the five *E. coli* genomes positive for *mcr-1* and *mcr-3* genes.

	CP131	CP55	CP61	CP66-6	CP8-3
Col(MG828)_1__NC_008486	0	91.603	0	0	0
Col(Ye4449)_1__FJ696405	0	0	0	0	81.026
Col156_1__NC_009781	0	98.026	0	0	0
ColKP3_1__JN205800	0	0	0	100	0
ColRNAI_1__DQ298019	0	89.256	0	0	0
IS1006	0	0	0	99.878	0
IS102	0	0	94.896	0	0
IS1203	0	95.42	0	95.42	0
IS1294	0	0	0	99.763	99.763
IS150	99.861	0	99.792	0	99.861
IS15DI	0	99.146	0	0	99.512
IS15DIV	0	100	0	0	0
IS186B	99.925	99.925	99.925	0	99.925
IS1A	90.625	0	90.625	0	98.958
IS1F	0	96.597	0	0	0
IS1H	0	98.56	0	98.429	0
IS1R	94.01	99.219	0	90.625	0
IS1SD	0	0	98.827	98.564	98.957
IS1X2	0	0	0	0	97.786
IS2	95.642	96.093	95.672	95.492	0
IS200	0	89.255	0	0	0
IS200C	0	0	0	99.577	0
IS26	99.878	0	99.634	100	99.616
IS3	99.921	99.921	99.921	99.921	0
IS30	99.836	99.836	99.918	99.545	99.836
IS3000	0	0	0	99.866	0
IS4321R	0	99.548	0	0	0
IS5	0	0	0	0	0
IS5075	0	99.623	0	0	0
IS609	0	97.597	97.426	97.654	97.712
IS6100	0	0	0	100	0
IS621	0	99.923	0	99.845	0
IS629	99.542	97.786	0	0	99.466
IS903B	95.845	0	98.297	89.225	96.975

IS911	94.052	0	0	0	0
ISAba1	0	0	0	0	0
ISApl1	99.907	0	99.907	99.907	99.907
ISCro1	98.111	0	0	0	0
ISCro3	0	0	0	90.756	0
ISEc1	0	96.897	96.406	96.664	95.279
ISEc12	99.961	0	0	0	0
ISEc17	0	0	0	0	99.921
ISEc23	0	96.327	0	0	0
ISEc26	0	98.677	98.511	98.677	0
ISEc31	0	92.732	0	0	0
ISEc32	0	100	0	0	0
ISEc39	0	99.62	0	0	0
ISEc5	90.495	0	0	0	0
ISEc8	0	99.14	85.003	0	85.003
ISErsp1	0	88.436	0	0	0
ISKpn26	96.739	0	0	0	99.582
ISSso1	0	87.215	0	0	0
ISSty2	0	0	81.445	0	0
ISSwi1	0	0	0	0	0
ISVsa3	0	0	0	100	0
ISVsa5	0	0	98.947	99.925	0
IncB/O/K/Z_2_GU256641	0	95.484	0	0	0
IncFIA(HI1)_1_HI1_AF250878	0	0	98.711	0	98.711
IncFIB(AP001918)_1__AP001918	98.827	98.507	0	97.678	0
IncFIB(K)_1_Kpn3_JN233704	0	0	98.929	0	98.929
IncFIB(pHCM2)_1_pHCM2_AL513384	0	0	0	0	0
IncFIC(FII)_1__AP001918	94.188	0	0	94	0
IncFII(pSE11)_1_pSE11_AP009242	0	94.34	0	0	0
IncFII_1__AY458016	0	100	0	100	100
IncHI1A_1__AF250878	0	0	0	0	0
IncHI1B(R27)_1_R27_AF250878	0	0	0	0	0
IncN_1__AY046276	0	0	100	0	100
IncQ1_1__HE654726	0	0	0	96.222	100
IncR_1__DQ449578	0	0	100	0	100

IncX1_1__EU370913	0	0	98.663	0	98.93
IncX3_1__JN247852	0	0	0	100	0
IncX4_1__CP002895	0	100	0	0	0
IncY_1__K02380	0	0	0	0	0
QnrS1_1_AB187515	0	100	0	100	100
QnrS2_1_JF261185	0	0	100	0	0
aac(3)-IId_1_EU022314	0	99.884	0	99.884	0
aac(3)-VIa_1_M88012	0	0	99.778	0	99.778
aadA1_1_X02340	0	0	89.5	89.5	89.625
aadA2_2_JQ364967	100	0	0	0	0
aph(3')-Ia_1_V00359	0	0	100	100	0
blaOXA-181_1_HM992946	0	0	0	100	0
blaTEM-1B_1_JF910132	100	100	100	99.884	100
catA1_1_V00622	0	99.848	0	0	0
cmlA1_1_M64556	0	0	99.921	99.921	99.841
dfrA12_1_AB571791	100	0	100	100	100
erm(B)_18_X66468	0	0	0	0	0
floR_2_AF118107	98.188	98.188	98.188	98.105	98.188
fosA_14_AB522970	0	0	0	91.367	0
lnu(F)_1_EU118119	0	0	0	100	0
mcr-1_1_KP347127	100	100	100	100	100
mcr3_NG_055505	0	99.831	100	99.831	99.831
mef(B)_1_FJ196385	0	0	100	0	100
mph(A)_2_U36578	99.675	99.636	0	99.566	0
strA_4_AF321551	0	99.876	0	0	0
strB_1_M96392	0	99.881	0	0	0
sul2_2_GQ421466	0	100	0	100	0
sul3_2_AJ459418	100	0	100	100	100
tet(A)_4_AJ517790	99.917	100	99.917	99.917	99.917
tet(M)_4_X75073	0	0	0	0	99.896
tet(M)_8_X04388	96.146	0	96.146	96.146	96.146

Table S3. Genetic characterization of *mcr-1* and *mcr-3* genes among the five strains in this study.

Strains	*mcr-1* location	*mcr-1* structures	*mcr-3* genes	*mcr-3* location	*mcr-3* structures

CP8-3	chromosome	IS*Apl1*-*mcr-1*-*∆pap2*-IS1294- IS*Apl1*	*mcr-3.5*	IncR plasmid	*qnrS1*-IS*Kpn19*-Tn*As2*-*mcr-3.5*-*dgkA*-IS*15*-Tn*As2*
CP55	IncX4 plasmid	*mcr-1-pap2*	*mcr-3.5*	IncFII plasmid	IS*4321R*-Tn*As2*-*mcr-3.5*-*dgkA*-IS*15*
CP61	chromosome	IS*Apl1*- *mcr-1*- *pap2*- IS*Apl1*	*mcr-3.1*	IncFIB plasmid	IS*Ec78*-Tn*As2*-*mcr-3.1*-*dgkA*-IS*Kpn40*
CP66-6	chromosome	IS*Apl1*- *mcr-1*-*∆pap2*-IS*1294*- IS*Apl1*	*mcr-3.5*	IncFII plasmid	*bla*$_{\text{TEM-1B}}$-Tn*As2*-*mcr-3.5*-dgkA-IS*26*
CP131	chromosome	IS*Apl1*- *mcr-1*- *pap2*- IS*Apl1*	*mcr-3.1*	IncFIB plasmid	*aac(3)-IId*-IS*1R*-Tn*As2*-*mcr-3.1*-*dgkA*-IS*Kpn40*

Table S4. The ratios of subclones losing *mcr-1* or/and *mcr-3* genes among the five *E. coli* strains during bacterial passages last for 28 days. P denotes the serial cultural passage day (1, 7, 14, 21 and 28). The denominators represent the average total colony numbers in the agar plates, and the numerators for the numbers of colonies losing the *mcr* genes confirmed by PCR. All the ratios in red show the passaged cultures with colonies losing *mcr* genes.

Strains	Loss of *mcr-1* without colistin					Loss of *mcr-3* without colistin				
	Clone_loss_P1	Clone_loss_P7	Clone_loss_P14	Clone_loss_P21	Clone_loss_P28	Clone_loss_P1	Clone_loss_P7	Clone_loss_P14	Clone_loss_P21	Clone_loss_P28
CP8-3	0/158	0/210	0/146	24/114	28/159	4/158	0/210	0/146	0/114	0/159
CP55	0/123	0/201	0/133	0/152	0/78	4/123	0/201	16/133	20/152	4/78
CP61	0/92	0/131	8/77	4/48	12/80	8/92	0/131	24/77	44/48	48/80
CP66-6	0/120	20/211	20/167	28/114	40/210	0/120	32/211	48/167	48/114	48/210
CP131	0/83	0/101	0/196	4/112	0/105	0/83	0/101	0/196	0/112	4/105
Strains	Loss of *mcr-1* with colistin					Loss of *mcr-3* with colistin				
	Clone_loss_P1	Clone_loss_P7	Clone_loss_P14	Clone_loss_P21	Clone_loss_P28	Clone_loss_P1	Clone_loss_P7	Clone_loss_P14	Clone_loss_P21	Clone_loss_P28
CP8-3	0/138	0/220	0/200	0/150	0/131	0/138	0/220	0/200	0/150	0/131
CP55	0/64	0/248	0/83	0/109	0/102	0/64	0/248	0/83	0/109	0/102
CP61	0/148	0/138	0/105	0/60	0/61	4/148	0/138	0/105	0/60	4/80
CP66-6	0/100	0/168	0/142	0/75	0/45	0/100	8/168	44/142	40/75	45/45
CP131	0/45	0/112	0/65	0/52	12/50	0/45	0/112	0/65	0/52	0/50

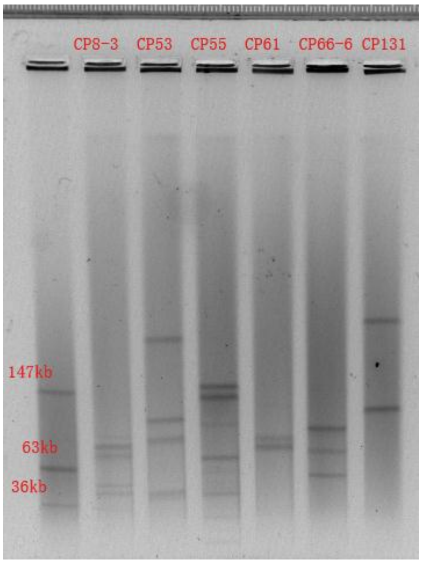

Figure S1. S1-PFGE of the original five *mcr*-bearing *E. coli* strains. The first lane indicates the plasmid weight markers (S1-nuclease digested 39R). CP53 was not included in this study.

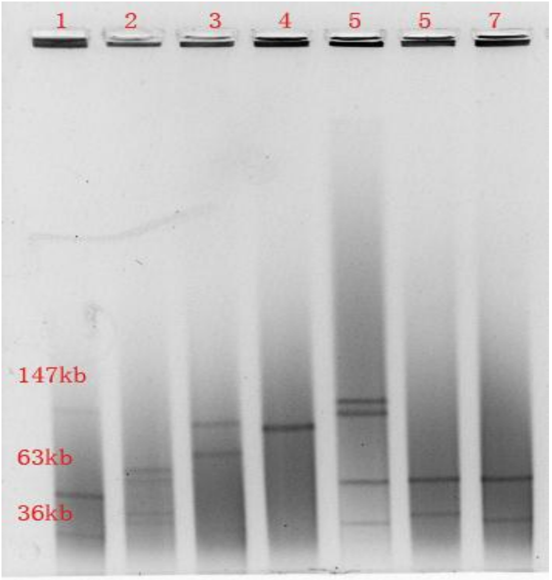

Figure S2. S1-PFGE of two *mcr*-bearing *E. coli* strains CP8-3 and CP55, and their transconjugants. Lane1: S1-nuclease digested 39R as a molecular weight marker; Lanes2: CP8-3 (donor), Lanes3: CP8-3-T (transconjugant), Lane5: CP55 (donor), Lane6: CP55-T1 (transconjugant), Lane7: CP55-T2 (transconjugant).

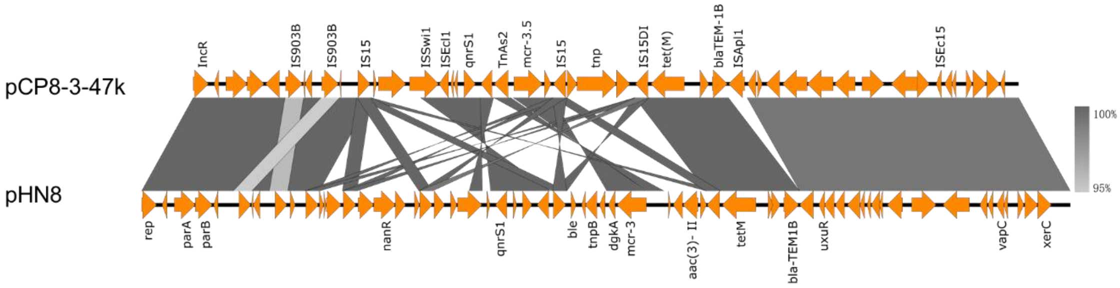

Figure S3. Linear alignment of two IncR *mcr-3*-bearing plasmids with similar backbone structures.

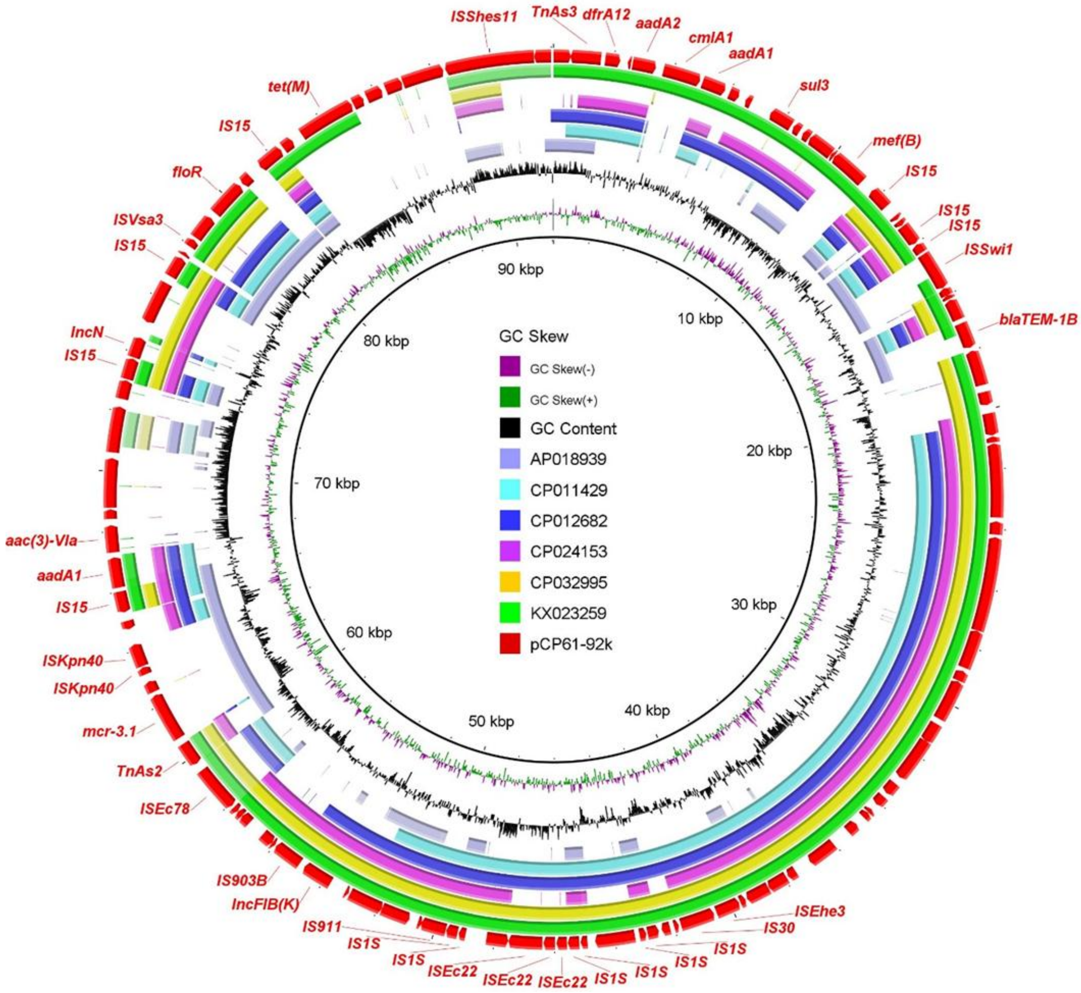

Figure S4. Circular alignment of *mcr-3.1*-bearing plasmid pCP61-92k with other similar plasmids in NCBI database.

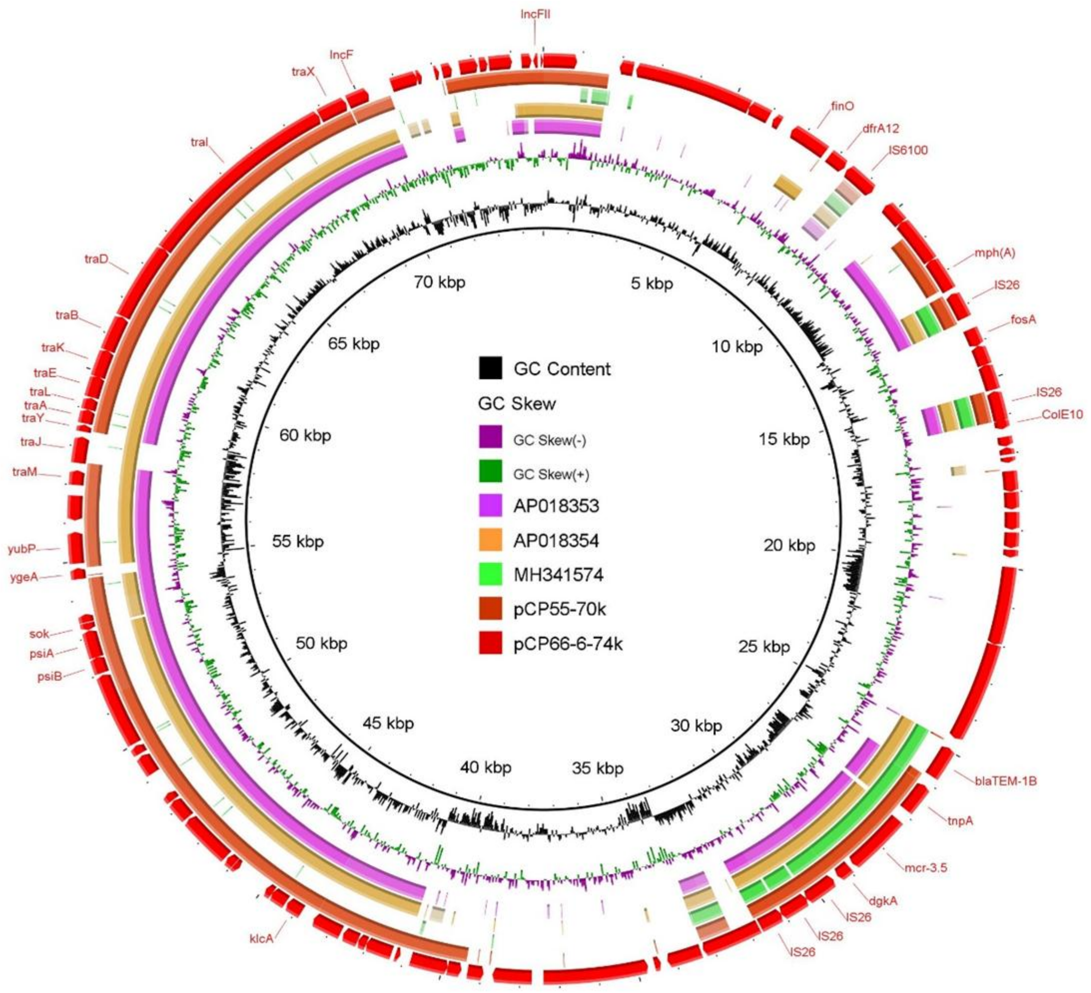

Figure S5. Circular alignment of *mcr-3.5*-bearing plasmid pCP66-6-74kb with other similar plasmids in this study and NCBI database.
